# Time-Dependent Effect of Hypoxia on Tumor Progression and Liver Progenitor Cell Markers in Primary Liver Tumors

**DOI:** 10.1371/journal.pone.0119555

**Published:** 2015-03-20

**Authors:** Eliene Bogaerts, Femke Heindryckx, Lindsey Devisscher, Annelies Paridaens, Yves-Paul Vandewynckel, Anja Van den Bussche, Xavier Verhelst, Louis Libbrecht, Leo A. van Grunsven, Anja Geerts, Hans Van Vlierberghe

**Affiliations:** 1 Gastro-enterology and hepatology, Ghent University, Ghent, Belgium; 2 Department of Medical Biochemistry and Microbiology, Uppsala University, Uppsala, Sweden; 3 Department of Pathology, University Hospital Ghent, Ghent, Belgium; 4 Liver Cell Biology Lab, Vrije universiteit Brussel, Brussels, Belgium; University of Medicine, Greifswald, Germany

## Abstract

**Background & Aims:**

Expression of liver progenitor cell (LPC) characteristics has been proposed as a negative prognostic marker in primary liver tumors. Hypoxia has been linked to activation of the Notch pathway which is responsible for activation and proliferation of LPCs and hypoxia-induced LPC activation has been shown in hepatocellular carcinoma. Our aim was to elucidate the time-dependent effects of hypoxia on the LPC niche in hepatocellular carcinoma which could aid in determining a safe time frame for use of hypoxia inducing therapies.

**Methods:**

We used dimethyloxaloylglycine to mimic a hypoxic reaction in mice by stabilizing hypoxia-inducible factor 1 alpha at three distinct time points in diethylnitrosamine induced hepatocarcinogenesis. LPC, metastasis and Notch pathway markers were determined by quantitative PCR and (immune)histochemistry (heamatoxillin-eosin, reticulin, Sirius red and cytokeratin 19 staining).

**Results:**

Activating the hypoxia inducible pathway early in hepatocarcinogenesis resulted in an increased incidence of both cholangioma and hepatocellular lesions, associated with high expression of LPC, metastatic and Notch pathway markers. Adversely, activating the hypoxic response during tumor development resulted in decreased incidence of hepatocellular lesions and increased cholangioma incidence, with an unaltered gene expression profile of LPC-, Notch pathway- and metastatic markers. A hypoxic insult at advanced stages of hepatocarcinogenesis severely increased the expression of LPC characteristics, however without increased expression of actors of the Notch pathway and metastatic markers and minor changes in incidence of hepatocellular and cholangioma lesions.

**Conclusion:**

Our results indicate that increased hypoxia at the onset of tumor development has detrimental effects on tumor progression; patients with HCC developed in a background of fibrosis/cirrhosis might therefore represent a more difficult treatment group. In contrast, hypoxia during tumor development appears to favor tumor outcome, highlighting the importance of early detection. Finally, hypoxia in advanced stages resulted in increased expression of LPC characteristics indicating poor outcome.

## Introduction

Primary liver tumors, especially hepatocellular carcinoma (HCC), often develop in a background of chronic liver disease, characterized by fibrosis and eventually cirrhosis. This process is accompanied by increased hypoxia, caused by sinusoidal capillarization and formation of fibrotic septa, increasing resistance to blood flow and thus decreasing oxygen delivery to liver cells [1]. In addition, fast growing liver tumors quickly outgrow the existing liver vascularization and newly formed intra-tumoral vessels are often structurally and functionally abnormal [2]. Ideally, applied anti-angiogenic treatment inhibits further extension of this poorly structured blood supply, depriving tumor cells of oxygen resulting in growth arrest [3, 4]. However, this intra-tumoral hypoxia, can also result in inhibition of prolyl hydroxylase domains (PHD), leading to stabilization of the hypoxia inducible factor 1 alpha (HIF-1α) resulting in transactivation of a plethora of genes such as the pro-angiogenic vascular endothelial growth factor alpha (Vegfa), and members of the glycolytic pathway such as glucose transporter 1 (Glut1) and phosphofructokinase (Pfk) aiding tumor cell survival [2, 4]. Therapy resistance to sorafenib has been linked to increased HIF signalization and anti-angiogenic treatment has been identified to cause increased local invasion and metastasis, worsening tumor progression [5–8].

Liver progenitor cells (LPCs) reside in the canals of Hering and are activated upon severe acute or chronic hepatic injury [9]. These bipotential progenitor cells proliferate and migrate towards the site of injury to replace hepatocytes and/or cholangiocytes and restore liver function. Interest in the role of LPCs in liver disease pathogenesis has recently expanded [9–15] and the knowledge that Notch and Wnt signaling drive LPC differentiation towards cholangiocytes or hepatocytes respectively has opened new perspectives into the regulation of hepatic cell differentiation[10, 15].

Several other pathways, including the HIF-1α-pathway have been linked to differential LPC behavior in liver disease and cancer [14]. For example: exposure of HCC cells to hypoxia significantly increased stem cell marker expression *in vitro* which could account for the observed dedifferentiation in tumors with low oxygen supply [16]. Interestingly, PHD_2_ haplodeficient mice, in which the HIF-dependent pathway is continuously activated, show increased cholangiocarcinoma (CC) burden, coinciding with increased expression of liver progenitor cell (LPC) markers after diethylnitrosamine (DEN) induced HCC induction [17]. Additionally, TACE treatment is also able to switch tumor phenotype from HCC to mixed HCC-CC, with increased expression of LPC markers, a more aggressive character and worse prognosis compared to HCC [18, 19].

Treatment induced hypoxia may thus increase the expression of stem/progenitor characteristics, which can mediate tumor progression, invasion, metastasis, therapy resistance, early post-operative recurrence and induce a phenotypic switch [5, 6, 8, 17–24]. Elucidating the time points in hepatocarcinogenesis at which activation of the hypoxic pathway has detrimental effects with respect to tumor outcome may allow us to anticipate and adapt current therapeutic strategies. Therefore, we assessed the time dependent consequences of elevated HIF signaling on tumor progression and LPC activation by using the PAN-PHD inhibitor dimethyloxaloylglycine (DMOG) in an orthotopic HCC mouse model.

## Materials and Methods

### Primary tumor induction and dimethyloxaloylglycine (DMOG) mediated activation of the HIF pathway

#### Ethics statement

All experiments were evaluated and approved by the Ghent University, faculty of health and medicine’s ethical commission for animal testing (ECD 12/57) and all efforts were made to minimize animal discomfort.

Weekly intraperitoneal (IP) DEN (Sigma—Aldrich, Bornem, Belgium) injections (35mg/kg) are known to induce neoplastic regions after 16 weeks, HCC nodules after 20 weeks and HCC with 100% penetrance from 25 weeks on [2]. For this study we administered DEN for 22 weeks in 5-week-old male 129S2/svPasCrl mice, control mice received weekly doses of saline equivalent to DEN counterparts.

**Fig 1:**
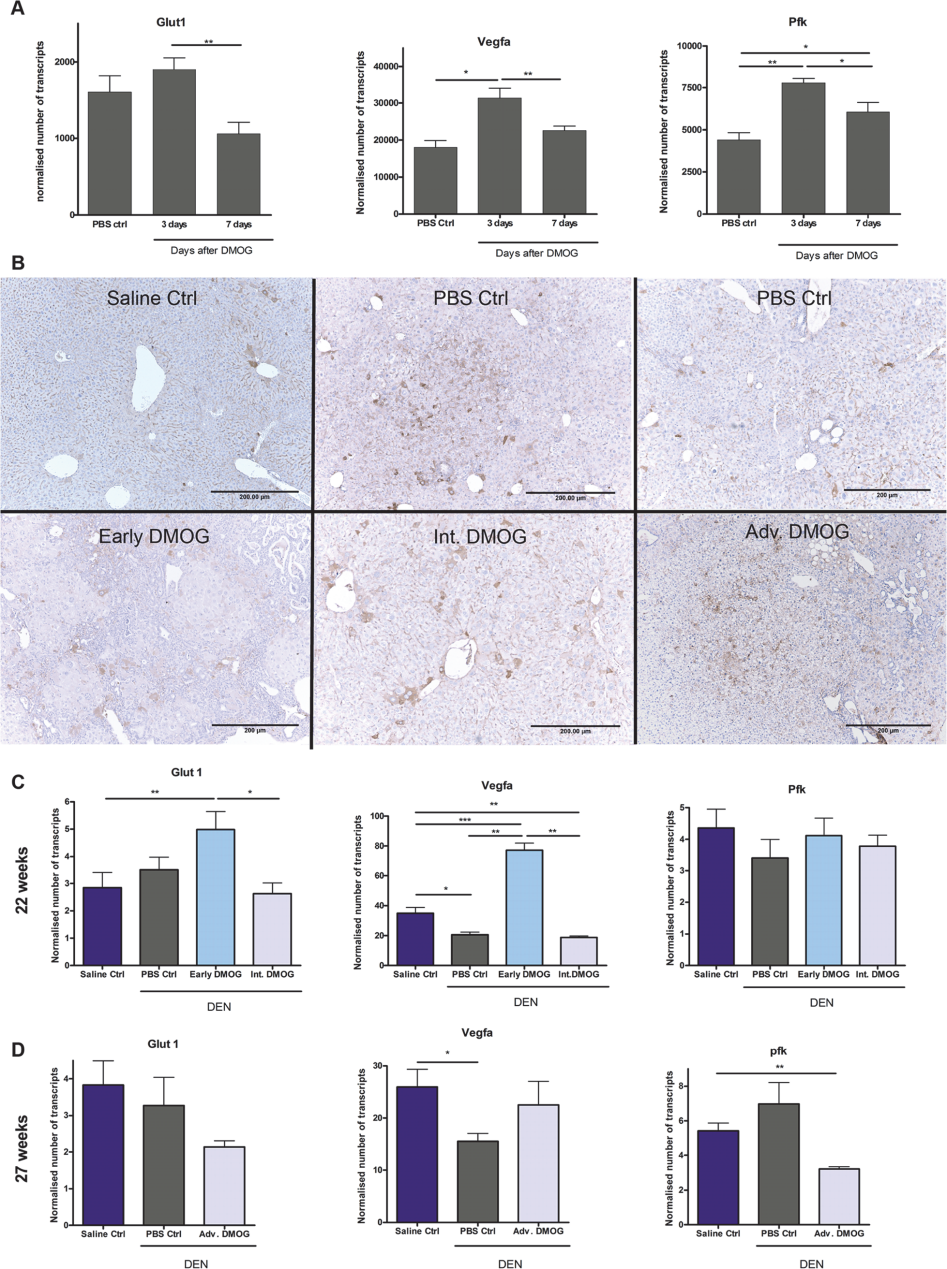
Hypoxia inducible factor expression. **A**: Expression of HIF-1α target genes, characteristic for stabilization of HIF-1α, 3 and 7 days after single DMOG injection **B**. Representative images of HIF-1α staining, showing HIF stabilization in all DEN groups, mostly located in and around cholangioma and HCC lesions and near portal areas. **C**. mRNA expression for HIF-1α markers. For animals sacrificed after 22 weeks; saline Ctrl: n = 8, PBS Ctrl: n = 6, Early DMOG: n = 8, Int DMOG: n = 6 and for animals sacrificed after 27 weeks; saline Ctrl: n = 8, PBS Ctrl: n = 7, Adv DMOG: n = 7. Scale bars: 200μm, *: p<0,05; **p<0,01 Hif-1α: hypoxia inducible factor 1 alpha, DMOG: dimethyloxaloylglycine, DEN: diethylnitrosamine.

Dose and interval of DMOG, which has been shown to induce HIF-1α stabilization [25], was first tested for its ability to effectively induce functional HIF-1α by measuring the transactivation of Vegfa, Glut1 and Pfk. Mice received a single IP DMOG injection (4,8mg/20g) followed by euthanasia after 3 and 7 days. Livers were removed and sections were lysed for RNA extraction and qPCR. Results showed that biweekly DMOG injections effectively induce HIF activation (Fig. 1A) and this treatment strategy was further applied.

DEN-treated mice received DMOG or PBS for five weeks at three different time points during tumor development: at early (1–5 weeks), intermediate (Int, 16–22 weeks) and advanced (Adv, 22–27 weeks) stages. For comparability between intermediate and advanced treatment groups and to reduce bias by acute DMOG effects, we chose to sacrifice mice from these groups 7 days after the final DMOG injection. saline control mice received DMOG from week 16 to 22 or from week 22 to 27 and were sacrificed after respectively 22 or 27 weeks

Scarification was preceded by anesthesia of the mice with isoflurane (Florene, Abbott, Hoofddorp, the Nederlands) in oxygen for weighting and blood sampling from the ophthalmic artery. After cervical dislocation, the liver was prelevated and weighed. Part of the liver was emerged in RNA later (Ambion, Gent, Belgium) and snap frozen. Remaining tissue was incubated in 4% phosphate buffered formaldehyde (KP4078.9010 Klinipath, Olen, Belgium) and imbedded in paraffin, as previously described [1, 2, 7].

### Immunohistological analyses

Hematoxilin-eosin (H&E) staining was performed as previously described [26] and sections were analyzed by a pathologist for general morphology and neoplasticity based on the following characteristics: enlarged cells with normal nucleus to cytoplasm ratio (n/c), small cells with increased n/c, enlarged pleomorphic nuclei, and binucleation.

Sirius red staining was performed as routinely described [26] to assess fibrosis which allows distinction between areas of ductular proliferation and cholangioma characterized by presence of typical cholangiofibrosis [7, 17].

Reticulin staining was performed to evaluate the presence of HCC nodules [26], which are absent for reticulin.

HIF-1α stabilization was evaluated through immunohistochemistry, using a rabbit anti—HIF-1α antibody (sc-10790, 1/400 in PBS, RRID: AB_2116990, Santa Cruz biotechnology, INC, California USA). Cytokeratin 19 (CK19) staining was performed to visualize structures of the cholangiocytic lineage, including LPCs, using monoclonal rabbit anti-CK19 (1/200 in TBS, ab133496, RRID:AB_11155282, abcam, Cambridge, UK). Epithelial cell adhesion molecule (Epcam) expression was examined using a goat polyclonal antibody raised against the transcriptionally active intracellular domain of Epcam (sc-23788, 1/300 in PBS, RIDD: AB_2098653, Santa Cruz biotechnology, INC, California, USA). LSAB- horseradish peroxidase-mediated visualization (K0690, DAKO, Heverlee, Belgium) was performed for all protocols. Overall immunoreactivity was calculated using Cell D software (Olympus Imaging Solutions, Münster, Germany) to assess increased expression of all cells of the cholangiocytic lineage. Since cholangiocytes organize in ductular structures and LPCs occur as singular cells, 5 portal areas were centered at a magnification of 400 and all CK19+ single cells were counted.

### Quantitative real time PCR (qPCR)

RNA was extracted from 20 mg of frozen liver tissue preserved in RNA-later, according to the manufacturer’s guidelines (Rneasy Mini Kit, Quiagen, Venlo, the Nederlands).

cDNA was obtained from 1μg RNA using the iScript cDNA synthesis kit (Bio-Rad, Nazareth-Eke, Belgium) and real time quantitative PCR (RT-qPCR) analyses were performed using the Lightcycler 480 Green I master mix (Roche, Vilvoorde, Belgium).

Primer sets are listed in S1 Table, their efficiency was calculated from the slope of a standard curve using the following formula: $E=10^{-\frac{1}{slope}}-1$. All reactions were run in duplicate and normalized to reference genes that showed stable expression in all samples. The comparative Ct method was used to determine the number of transcripts.

### Statistics

Data were analyzed using SPSS21 software (IMB corp, Armonk NY, USA). Kolmogorov-Smirnov test was used to test for normality. Student’s T- test was then performed in case of normality; the Mann-Whitney-U test was used for not normally distributed data. P-values ≤0,05 where considered significant. All data are presented as average ±SEM.

## Results

### Time dependent effect of HIF stabilization on DEN-induced tumorigenesis

We first analyzed whether 4,8mg/20g biweekly or weekly DMOG injections are required to maintain stable activation of the HIF-pathway in livers, by performing qPCR analysis of HIF sensitive genes like Vegfa, Glut1 and Pfk. There was an increased expression of HIF sensitive genes, for at least 3 but not 7 days, significant for Vegf and Pfk after DMOG induction, compared to PBS control (Fig. 1A). Further treatment regimes were therefore carried out by biweekly DMOG injections.

**Fig 2:**
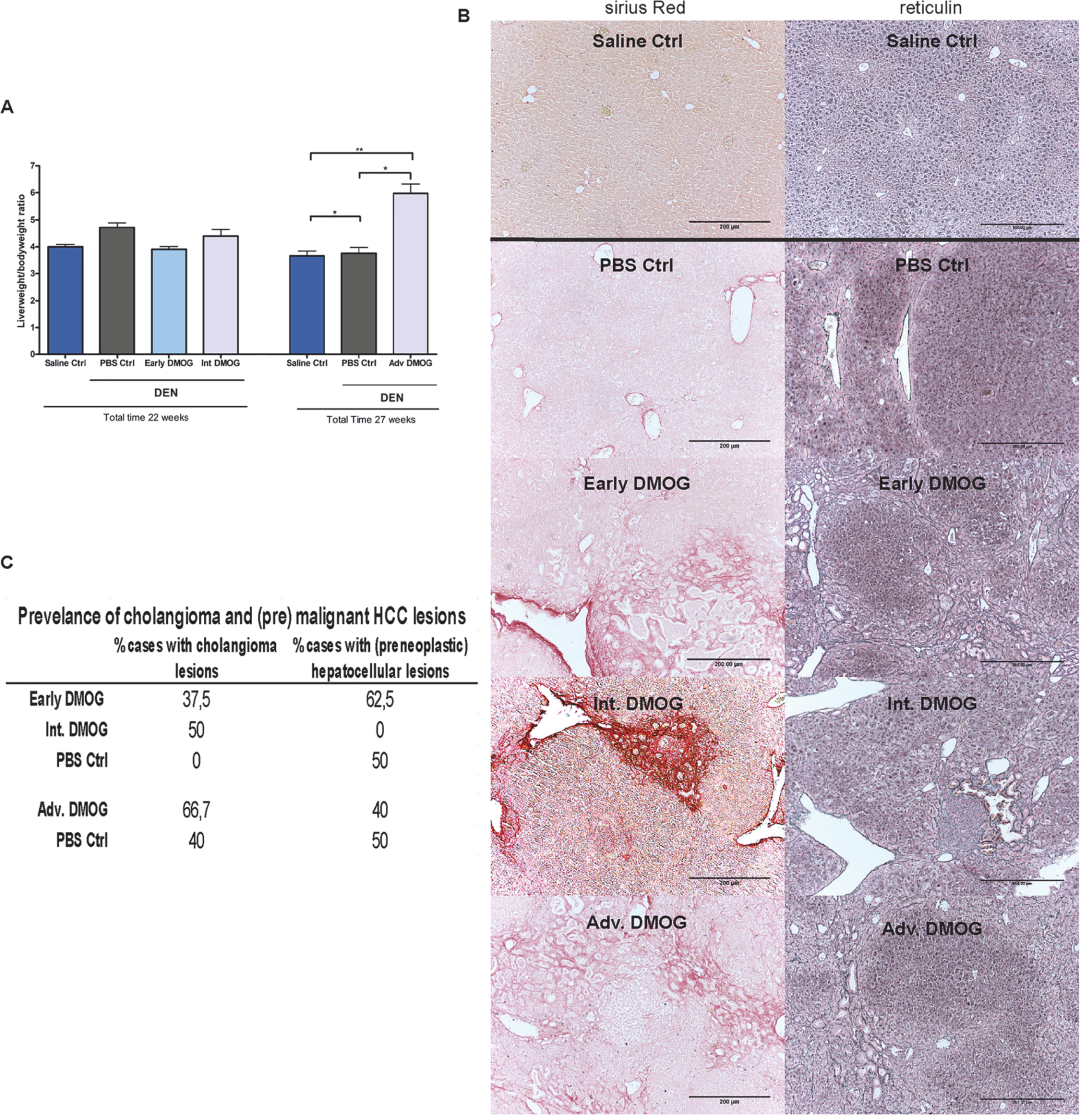
General parameters. **A**: Liver/ bodyweight ratios for all groups **B**: Prevalence of cholangioma and hepatocellular lesions, showing percentage of mice showing one or more cholangioma or (premalignant) HCC lesions **C:** Representative images of Sirius red and reticulin staining showing cholangioma lesions in all DMOG groups except for PBS control after 22 weeks, and HCC lesions in all DEN groups except for the Int. DMOG group. Scale bars: 200μm, *: p<0,05; **p<0,01. DMOG: dimethyloxaloylglycine; DEN: diethylnitrosamine

To assess the effect of HIF stabilization early on in tumorigenesis, DMOG was administered during the first 5 weeks of DEN treatment (Early). Samples were taken after 22 weeks, early DMOG treatment had no effect on relative liver weight (Fig. 2A). Sirius red staining revealed cholangioma formation in 37,5% and reticulin staining showed premalignant HCC lesions in 62,5% of mice compared to respectively 0 and 50% of the mice receiving PBS (Fig. 2B, C).

To evaluate the activation of the hypoxic pathway during intermediate stages, DMOG was injected from week 16 to week 22 (intermediate, Int.) during DEN induction. Samples were taken at the end of week 22, Int. DMOG also did not influence relative liver weight (Fig. 2A). Sirius red and reticulin staining in Int. DMOG-treated animals showed cholangioma formation in 50% of DMOG injected mice and no HCC lesions, compared to no cholangioma lesions and 50% HCC lesions in PBS control mice (Fig. 2B, C).

For the effect of HIF stabilization after the final DEN injection, during tumor growth, DMOG (or PBS) was administered from week 22 to 27 (advanced, Adv.) and samples were taken fter27 weeks. Adv. DMOG resulted in a significantly increased relative liver weight compared to PBS counterparts (Fig. 2A). Sirius red staining showed cholangioma lesions in 50% of PBS and 66,7% of DMOG induced animals and reticulin staining showed HCC lesions in 40% of DMOG and 50% of PBS treated livers (Fig. 2B, C).

This suggests that, Early Int. and Adv. DMOG result in increased cholangioma formation and Early DMOG even increases HCC formation, while Int. DMOG inhibits HCC formation.

Immunohistochemistry and qPCR analysis were then performed to assess HIF-1α stabilization and activity after Early, Intermediate and Advanced DMOG treatment. While HIF-1α immunopositivity was limited in saline control livers, in DEN treated livers there was some cytoplasmic presence of the HIF protein in hepatocytes and cholangiocytes around the portal area, but immunopositivity was mostly observed in and around hepatocellular and cholangioma lesions (Fig. 1B).

HIF-1α activity was determined through qPCR analysis for downstream HIF target genes. As expected, there was no increased expression in Int.—and Adv. DMOG groups compared to their PBS control groups (Fig. 1C). Strangely, these DEN groups also show no increased or even a decreased expression of HIF-dependant genes compared to saline control. However, we do see significantly increased mRNA expression of HIF target genes Glut1 and Vegfa in DEN livers after Early DMOG treatment compared to all other groups(Fig. 1C).

### Time dependent effect of hypoxia on LPC characteristics in DEN treated mice

**Fig 3:**
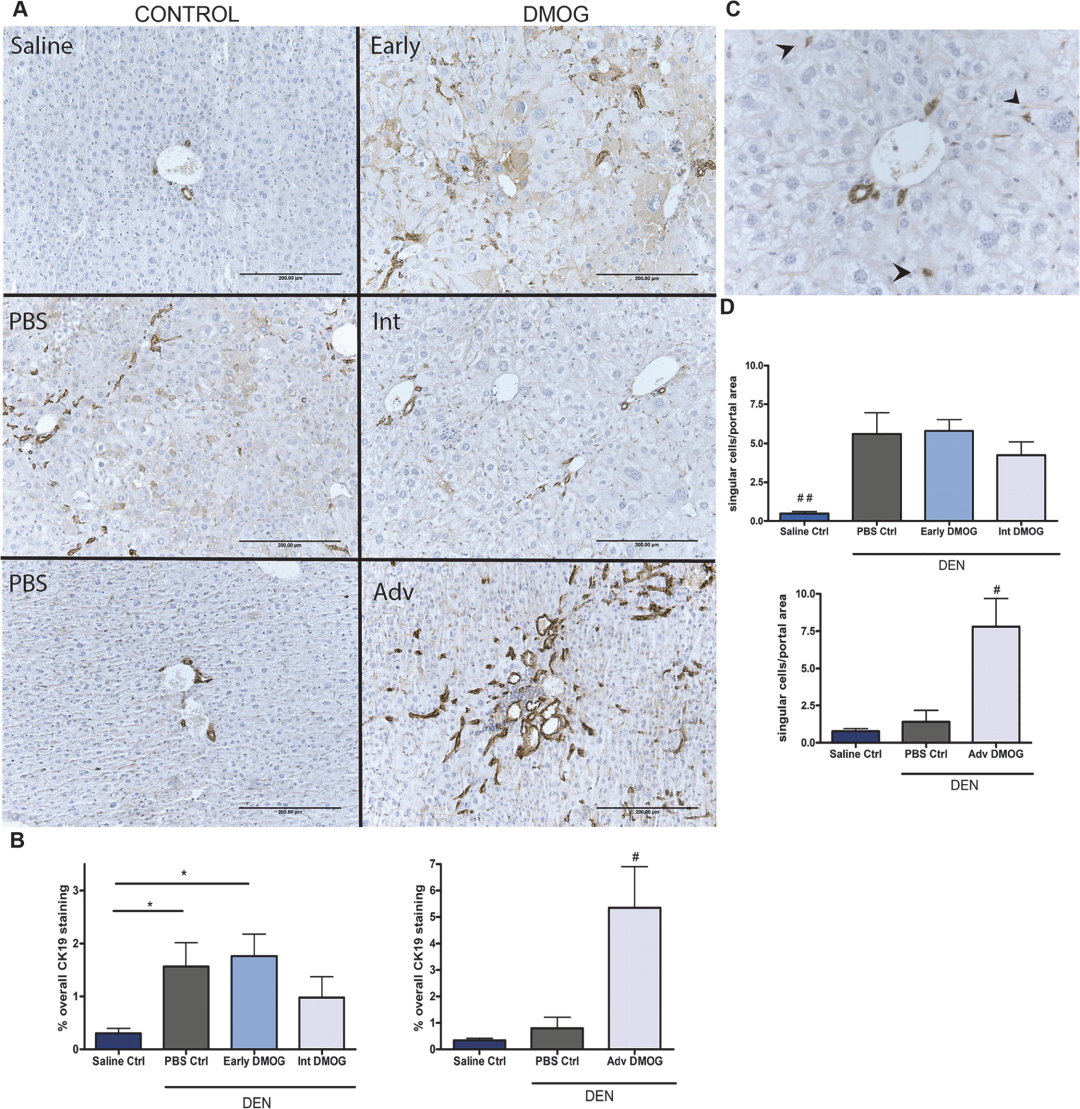
Cytokeratin 19. **A:** Representative images of overall CK19 staining, early DMOG and PBS counterparts also show cytoplasmatic staining in hepatocytes **B:** quantified % of overall CK19 staining after 22 and 27 weeks **C:** Image of portal area at 400x magnification as used to count single cells, arrowheads point to single cells **D:** Average number of single cells per portal area for each group. Scale bars: 200μm, *p<0,05, **p<0,01. #p<0,05, ##p<0,01 compared to all other groups. CK19: cytokeratin 19; DMOG: dimethyloxaloylglycine

Tumor sections were analyzed for overall CK19 immunopositivity, which is a marker for biliary epithelial cells, including LPCs. All animals receiving DEN showed increased immunopositivity for CK19 after 22 weeks compared to saline controls. (Fig. 3A and B left graph). Mice treated with DMOG at advanced stages showed significantly enhanced CK19 immunopositivity compared to PBS control (Fig. 3A and B right graph). CK19+ single cells (Fig. 3C) were numerous in livers of DEN treated groups compared to saline controls at 22 weeks (p<0,05), but no significant difference was seen between treatment regimes (Fig. 3D upper graph). DMOG treatment from week 22–27 did however result in a significant increase in CK19+ single cells compared to both PBS and saline control groups (Fig. 3D lower graph).

**Fig 4:**
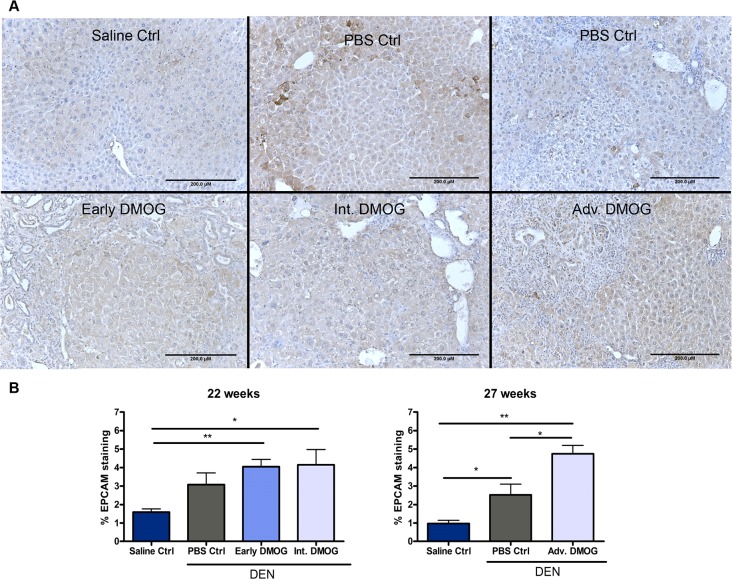
Epithelial Cell Adhesion Molecule. **A**. quantified % of Epcam intracellular domain staining after 22 and 27 weeks **B**. representative images of Epcam staining showing presence around cell membranes of hepatocytes and in cytoplasm of cholangiocytes in saline control livers (upper left). In DEN treated livers, cytoplasmic expression in hepatocytes around portal areas, cholangioma -and hepatocellular lesions was increased. Scale bars: 200μm *:p<0,05; **p<0,01. Epcam: epithelial cell adhesion molecule; DEN: diethylnitrosamine

Next, we examined sections for Epcam immunopositivity, which is a marker for biliairy epithelial cells, including LPCs[27] as well as tumor cells[28]. In saline control mice, staining was limited to cholangiocytes and some membranous staining of hepatocytes (Fig. 4A). In DEN treated mice immunopositivity was seen in the cytoplasm of hepatocytes, mostly around portal areas and in regions containing cholangioma -and hepatocellular lesions (Fig. 4A). These results were in line with those of CK19, with a positive trend to increased Epcam expression for all DEN mice, significant for all DMOG groups and for mice that received PBS from week 22 to 27 compared to saline control (Fig. 4B). Futhermore, Adv. DMOG treatment also resulted in a significantly increased Epcam immunopositivity compared to PBS control after 27 weeks (Fig. 4B).

**Fig 5:**
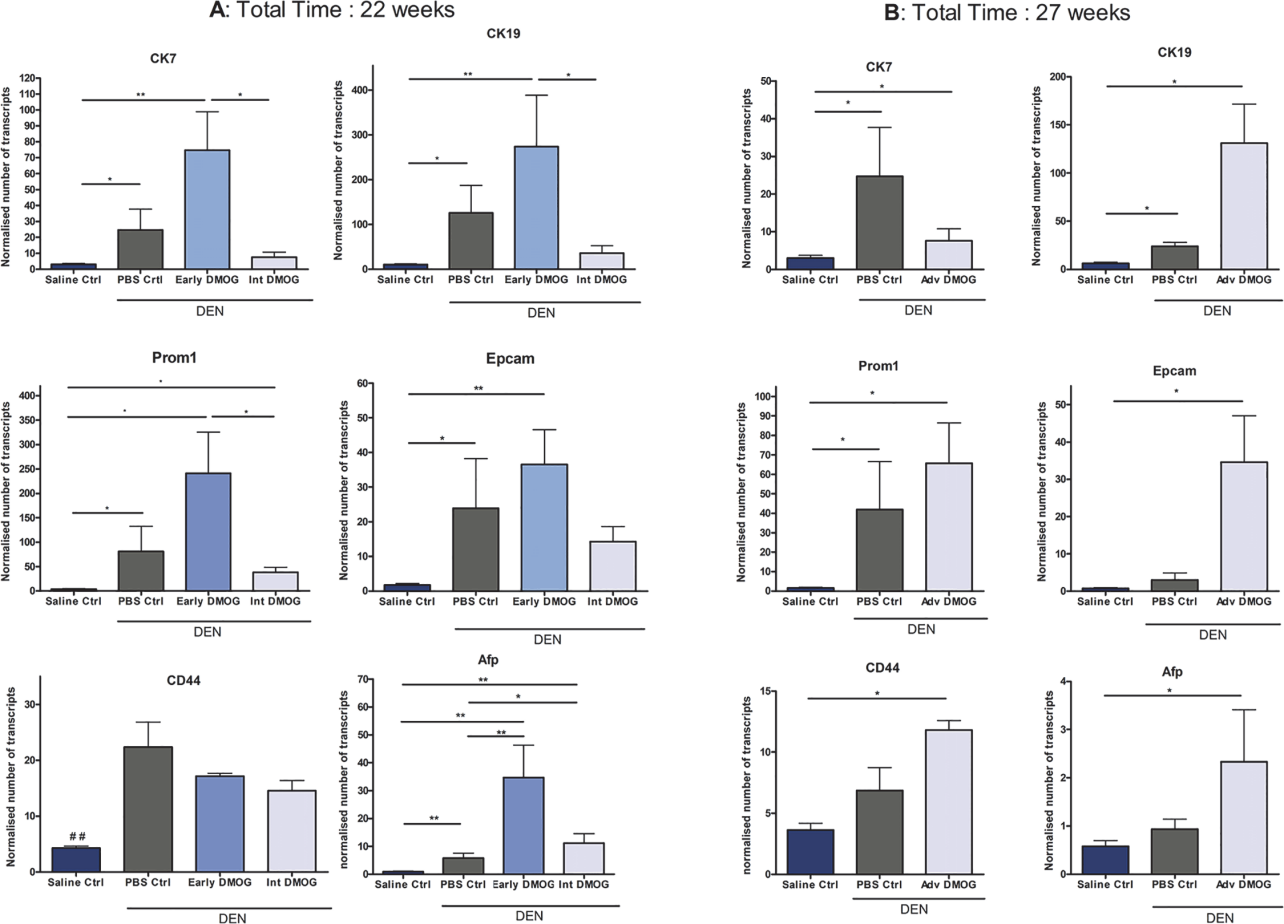
mRNA expression of LPC markersA: mRNA expression of LPC markers after early and Int. DMOG **B:** mRNA expression of LPC markers after Adv. DMOG. *:p<0,05; **p<0,01, ##<0,01. DMOG: dimethyloxaloylglycine; LPC: liver progenitor cell

Since progenitor cell markers have been proposed as markers of poor prognosis in HCC, we next examined the mRNA expression of liver progenitor cell markers cytokeratin 7 (CK7), CK19, CD44, alpha-fetoprotein (Afp), Epcam and prominin1 (Prom1). In DEN mice receiving DMOG at early stages, there was a non-significant increased expression of CK7, CK19, Epcam and Prom1, compared to PBS control, wich was not seen in mice treated with DMOG at intermediate stages (Fig. 5A). Suggesting a protective role for Int. DMOG and a previously unreported effect of early hypoxia. Afp expression was significantly increased after both early and intermediate DMOG treatment compared to PBS control (Fig. 5A).

Adv. DMOG resulted in a non-significant increase of all markers, except for CK7 where a non-significant decreased expression was seen, compared to PBS control. Both DEN groups showed increased expression of LPC markers compared to saline control (all significant for Adv. DMOG group, significant for Prom1, CK7 and CK19 for PBS group), (Fig. 5B).

**Fig 6:**
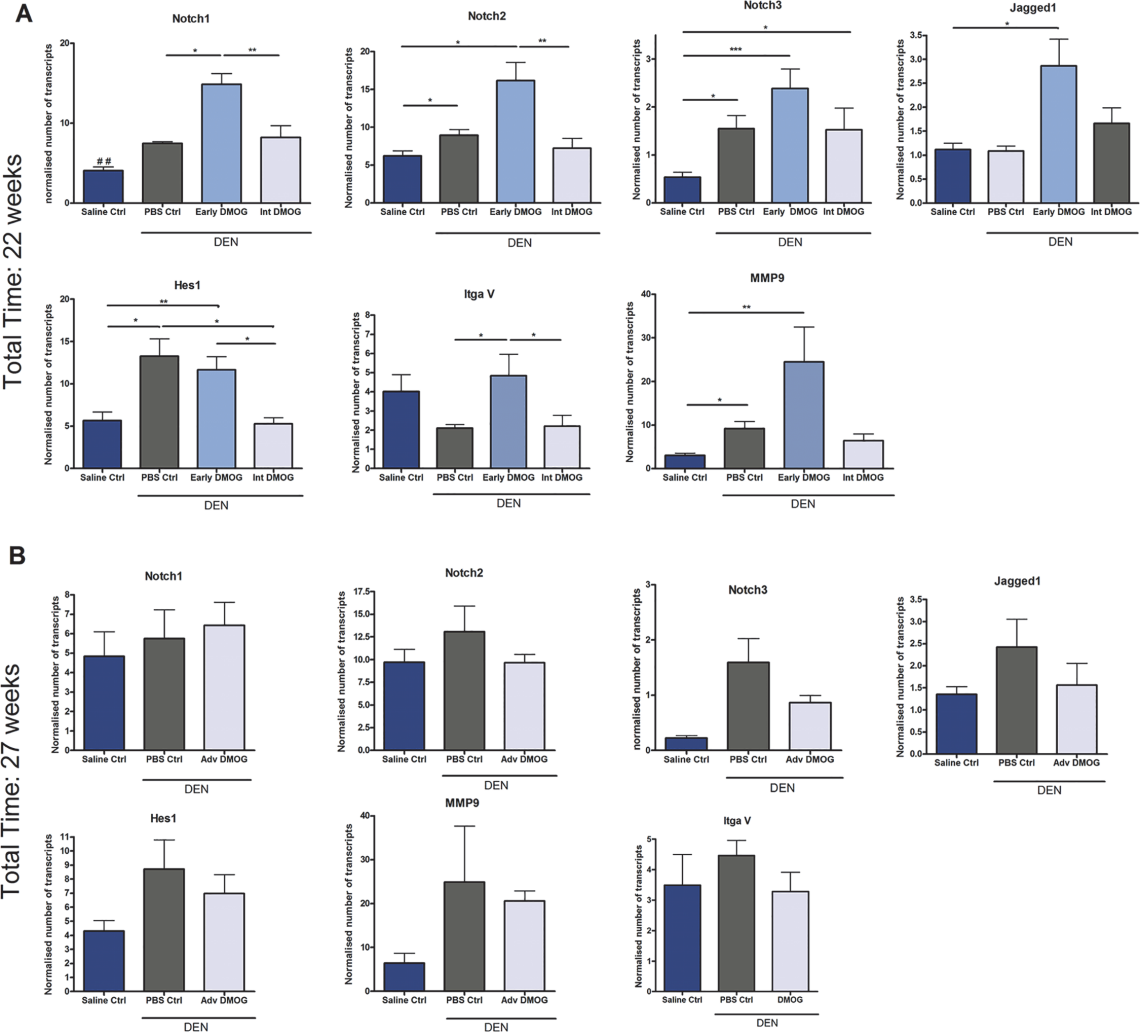
mRNA expression of Notch and metastasis markers. **A:** mRNA expression of Notch and metastasis markers after early and Int. DMOG **B:** mRNA expression of Notch and metastasis markers after Adv. DMOG in DEN induced hepatocellular carcinoma. *p<0,05; **p<0,01, ## p<0,01 compared to all other groups. DMOG: dimethyloxaloylglycine; DEN: diethylnitrosamine

Since Notch signaling is known to be involved in the differentiation of LPCs to cholangiocytes, and has also been suggested to mediate hypoxia induced therapy resistance and increased invasion/metastasis [14], we examined mRNA levels of Notch 1, Notch 2 and Notch3 receptors, the ligand Jagged1 and main target gene: hairy enhancer of split 1 (Hes1); and matrix metalloproteinase 9 (Mmp9) and Integrin alpha 5 (ItgaV) as markers for metastasis [29]. Interestingly, Notch and metastasis markers were only up-regulated in mice that received DMOG at early stages (Fig. 6). Table 1 summarizes the main findings for all groups.

**Table 1:** Summary of groups and major findings.

Timing DMOG	Early	intermediate	advanced
	week 1–5	week 16–22	week 22–27
DEN induction	22 weeks		
Total time	22 weeks		27 weeks
DEN control group	22w DEN +		22w DEN +
	PBS (week16–22)	PBS (week 22–27)
**General parameters**			
relative liver weight	⇵	⇵	**↑** *
	1,039 ± 0,02798	1,167 ± 0,06543	1,321 ± 0,07186
**Tumor type**			
Hepatocellular lesions	**↑**	↓	↓
Cholangioma	**↑**	**↑**	**↑**
**Liver progenitor cell markers**			
CK19+ single cells	⇵	↓	**↑** *
	1,036 ± 0,1279	0,7589 ± 0,1511	4,179 ± 1,012
CK19 mRNA	**↑**	↓	**↑**
	2,172 ± 0,9123	0,2844 ± 0,1336	5,711 ± 1,423
CK7 mRNA	**↑**	↓	↓
	3,020 ± 0,9846	0,3080 ± 0,1281	8,932 ± 2,806
Prom1 mRNA	**↑**	↓	**↑**
	2,973 ± 1,044	0,4728 ± 0,1257	1,972 ± 0,4273
Epcam mRNA	**↑**	↓	**↑**
	1,337 ± 0,4107	0,5967 ± 0,1828	14,29 ± 3,995
CD44 mRNA	↓	↓	**↑**
	0,8260 ± 0,1985	0,6499 ± 0,08237	4,075 ± 1,909
Afp mRNA	**↑** **	**↑** *	**↑**
	5,978 ± 2,008	1,912 ± 0,5875	2,913 ± 1,338
**Notch markers**			
Notch1 mRNA	**↑** *	⇵	**↑**
	2,096 ± 0,02613	1,185 ± 0,08566	1,460 ± 0,2858
Notch2 mRNA	**↑**	⇵	⇵
	1,807 ± 0,2696	0,8063 ± 0,1477	0,8674± 0,05704
Notch3 mRNA	**↑**	⇵	↓
	1,543 ± 0,2627	0,9848 ± 0,2933	0,6119 ±0,09868
Jagged1 mRNA	**↑**	**↑**	↓
	2,633 ± 0,5159	1,529 ± 0,2996	0,8272 ± 0,2529
Hes1 mRNA	⇵	↓*	⇵
	0,8795 ± 0,1181	0,4431 ± 0,04125	0,9004 ± 0,2051
**Metastatic markers**			
MMP9 mRNA	**↑**	⇵	⇵
	2,688 ± 0,8184	0,8170 ± 0,2145	0,8541 ± 0,1239
ItgaV mRNA	**↑** *	⇵	⇵
	3,063 ± 0,9327	0,9311 ± 0,2445	0,7502 ± 0,3067

First row: ⇵: No change compared to DEN control, ↑: Increase compared to DEN control, ↓: Decrease compared to DEN control. Second row: fold changes ±Standard deviation, compared to PBS control.

*: p<0,05

**: p<0,01

## Discussion

In the present study, we show that pan-PHD inhibition in early and advanced stages of hepatocarcinogenesis induces increased expression of LPC characteristics, while PHD inhibition in intermediate stages has a tendency to decrease the expression of LPC characteristics. Furthermore, the early, but not intermediate or advanced-stage HIF-1α stabilization, concurred with increased expression of actors of the Notch pathway and metastatic markers. These results indicate an important time- dependent effect of hypoxic stimuli in HCC and a previously undescribed detrimental delayed effect of an early hypoxic event on tumor development.

Currently LPCs are being intensively studied for their role in various liver diseases, and have recently also been implicated in the pathogenesis of primary liver tumors. Increased expression of LPC characteristics serves as a marker for poor prognosis [21, 23, 24, 30]. Moreover, since LPCs highly express multi drug resistance proteins, they are also implicated in therapy resistance [31]. Furthermore, different studies have shown a phenotypic switch from HCC to HCC-CC following hypoxic stimuli, coinciding with increased expression of progenitor cell markers [6, 17, 18]. Since activation of the hypoxic pathway could alter LPC behavior in hepatocellular carcinoma, we studied the effect of increased activation of HIF on DEN induced hepatocarcinogenesis.

We used DMOG to induce the hypoxic response, aimed at mimicking oxygen deprivation at different time points in tumor development. The early HIF stabilization reflects patients with chronic liver disease, characterized with fibrotic strands and hypoxia prior to tumor development. Additionally, early HIF stabilization may also mimic hypoxia-inducing treatment strategies affecting recurrent tumor behavior. The intermediate DMOG induction relates to patients undergoing anti-angiogenic treatment for early stage cancer. Lastly, the group receiving DMOG at advanced stages resembles patients receiving treatment for advanced stage HCC.

Immunohistochemistry for HIF-1α expresssion showed few hepatocytes expressing HIF in saline groups, and cells expressing the HIF protein in DEN groups were mostly residing in the portal area, and in and around hepatocellular- and cholangioma lesions, coinciding with CK19 and Epcam immunopositive regions.

Our aim was not to show HIF-1α stabilization following DMOG at specific time points but to evaluate its effect on tumorigenesis and LPC activation on the long term. We therefore chose to euthanize mice 7 days after the final DMOG injection which is confronted with an attenuation of HIF-1α stabilization and transcriptional activation as shown in by the single DMOG injection experiment. Activation of the HIF pathway in DEN groups was examined through qPCR analysis of HIF-1α target genes, no difference could be detected between intermediate and advanced DMOG and their respective PBS controls, confirming that acute effects of PHD inhibition were eliminated by euthanizing animals 7 days after the final DMOG injection.

Strangely, saline groups had an equal Glut1 and Pfk and even an increased Vegfa mRNA expression compared to intermediate and advanced DMOG groups and their PBS controls. Since saline mice had also received DMOG, possibly a variety of feedback loops could be differentially regulated in DEN compared saline mice [32, 33], which should be further investigated.

Interestingly, the DEN group that received DMOG at early stages, did show significantly increased expression Vegfa and Glut1 compared to other groups. HIF induction early in DEN- induced hepatocarcinogenesis also caused increased formation of cholangioma and HCC lesions as well as a massive upregulation of LPC features and metastatic markers on the RNA level, compared to groups that received DMOG or PBS at intermediate stages. This massive delayed effect of hypoxia has not previously been described and indicates that early hypoxia could readily prepare cells for later tumor growth and growth—induced hypoxia, resulting in tumors with a more aggressive phenotype.

Thus, monitoring the extent to which the hypoxic pathway is activated after hypoxic treatment for recurring tumors or as a result of inflammation and fibrosis in chronic liver disease could have prognostic value when these patients (re)develop HCC later on. Indeed, there is evidence of a phenotypic switch in tumors recurring after TACE, which induces a massive hypoxic response [18, 19].

Mice receiving DMOG during tumor development (at intermediate stages) displayed no HCC lesions and no altered expression of LPC or metastatic markers compared to PBS control mice. While the increased formation of cholangioma lesions should be monitored, these benign intrahepatic bile duct adenomas usually do not require treatment [34]. Taken together, this could point to a safe therapeutic window for hypoxia inducing treatment after early detection.

Administering the pan-PHD inhibitor DMOG in advanced stages of tumor development resulted in a significantly increased relative liver weight, a slight decrease in HCC and a minor increase in cholangioma lesions coinciding with a significantly increased expression of LPC markers and number of CK19+ single cells. This observation is in line with previous reports showing that treatment induced hypoxia is linked to an increased expression of stem/progenitor characteristics [16, 35, 36]. However, while this hypoxia-induced LPC signature did not coincide with increased expression of metastatic markers, HCC lesions with a cholangiocytic signature have been linked to poor prognosis and early recurrence [20–24, 30, 37]. Furthermore, while the increased relative liver weight compared to PBS counterparts is at least partly caused by the increased amount of cholangioma and its accompanying cholangiofibrosis, it could also be a sign of increased tumor burden.

Notch signalization is involved in LPC proliferation and pushes LPC differentiation towards cholangiocytic structures. Since we observed an increased incidence of cholangioma lesions in DEN- livers after a hypoxic insult, RNA expression of Notch related genes was analyzed. Increased mRNA expression of actors of the Notch pathway was seen in livers of mice receiving DMOG early in hepatocarcinogenesis coinciding with high expression of LPC- and metastatic markers.

This suggests a role for Notch- mediated increased proliferation of LPCs and differentiation towards cholangiocytes in the pathogenesis of HCC after early hypoxic stimuli, thus contributing to the development or recurrence of aggressive, more invasive tumors with a mixed phenotype. Indeed, pharmacological inhibition of the Notch pathway has already been proven to be effective in reducing the amount of chemo-resistant cancer stem cells in breast and colon cancer [38, 39]. Surprisingly, DMOG administration at both intermediate and advanced stages did not lead to increased expression of actors of the Notch pathway.

While CK19 and CK7 positive liver tumors have been proposed to be progenitor cell derived [37], *in vitro* experiments have shown that HCC cells are capable of trans differentiating towards a cholangiocytic phenotype [40, 41]. The fact that the stem cell marker Prom1 is only marginally up-regulated compared to the pronounced cholangiocytic markers CK7 and CK19, and there does not appear to be any Notch involvement in tumors undergoing hypoxia at advanced stages, might reflect this HCC trans-differentiation rather than LPC involvement. However, while whole liver analysis showed no Notch- pathway activation, individual cell populations should be analyzed for more clarity on Notch involvement.

The present study underlines that early hypoxic stimuli have detrimental effects on tumor progression with an increased expression of poor prognostic markers later on. Activation of the HIF pathway at advanced stages of tumorigenesis resulted in severely increased expression of LPC characteristics without Notch activation. Hypoxic treatment at intermediate stages of DEN induced hepatocarcinogenesis appears to have the least detrimental effect on tumor progression and reflects the advantages of early tumor diagnosis with most favorable treatment options/effects.

## Supporting Information

S1 Table Genes and primersets. (PDF)
