# Supplementary material for: Time-Dependent Effect of Hypoxia on Tumor Progression and Liver Progenitor Cell Markers in Primary Liver Tumors
Source: PLoS One. 2015 Mar 20;10(3):e0119555. doi: 10.1371/journal.pone.0119555 (PMC4368520; doi:10.1371/journal.pone.0119555)
Supplement: S1 Table — (PDF) [file pone.0119555.s001.pdf]

Table SI: Genes and primersets

Gene ID	short	Full name	Forward primer	Reversed primer	Marker
14433	GAPDH	glyceraldehyde 3 phosphate dehydrogenase	CATGGCCTTCCGTGTTCCTA	GCGGCACGTCAGATCCA	Reference
15288	HMBS	hydroxymethyl-bilane synthase	AAG GGC TTT TCT GAG GCA CC	AGT TGC CCA TCT TTC ATC ACT G	Reference
15452	HPRT	hypoxanthine guanine phosphoribosyl transferase	GTT AAG CAG TAC AGC CCC AAA	AGG GCA TAT CCA ACA ACA AAC TT	Reference
66945	SDHA	succinate dehydrogenase complex, subunit A	CTTGAATGAGGCTGACTGTG	ATCACATAAGCTGGTCCTGT	Reference
11576	AFP	alpha – fetoprotein	AGCTTCCACGTTAGATTCCTCC	ACAAACTGGGTAAAGGTGATGG	LPC
16669	KRT19	Cytokeratin 19	GTTCAGTACGCATTGGGTCAG	GAGGACGAGGTCACGAAGC	LPC
110310	CK7	Cytokeratin 7	AGGAGATCAACCGACGCAC	CACCTTGTTCGTGTAGGCG	LPC
12505	CD44	CD44 antigen	tcgatttgaatgtaacctgccg	CAGTCCGGGAGATACTGTAGC	LPC
19126	PROM1	Prominin 1	CTCCCATCAGTGGATAGAGAACT	ATACCCCCTTTTGACGAGGCT	LPC
17075	EPCAM	Epithelial cell adhesion molecule	GCGGCTCAGAGAGACTGTG	CCAAGCATTTAGACGCCAGTTT	LPC
16410	ITGAV	Integrine alpha 5	CAATTGCTGCTCCCTATGGT	GATTTGAGATGGCACCGAAT	Metastasis
17395	MMP9	Matrix metalloproteïnase 9	GAGACGGGTATCCCTTCGAC	TGACATGGGGCACCATTTGAG	Metastasis
16449	JAG1	Jagged 1	ATGCAGAACGTGAATGGAGAG	GCGGGACTGATACTCCTTGAG	NOTCH
18128	NOTCH1	Notch 1	GATGGCCTCAATGGGTACAAG	TCGTTGTTGTTGATGTCACAGT	NOTCH
18129	NOTCH2	Neurogenic locus notch homolog protein 2	ATGTGGACGAGTGTCTGTTGC	GGAAGCATAGGCACAGTCATC	NOTCH
18131	NOTCH3	Neurogenic locus notch homolog protein 3	AGTGCCGATCTGGTACAAGTT	CACTACGGGGTTCTCACACA	NOTCH
15205	HES1	Hairy enhancer of split 1	ACGTGCGAGGGCGTTAATAC	ACGTGCGAGGGCGTTAATAC	NOTCH
22339	VEGFa	Vascular endothelial growth factor A	ACTCGGATGCCGACACGGGA	CCTGGCCTTGCTTGCTCCCC	Hypoxia
20525	Glut1	Glucose transporter 1	GCT TAT GGG CTT CTC CAA ACT	GT GAC ACC TCT CCC ACA TAC	Hypoxia
18642	Pfk	Phosphofructokinase	GCCGGCTCAGTGAGACAAG	TGGCACCTTCAGCAACAATG	Hypoxia
